# The Effect of Relative Pubertal Maturation and Perceived Popularity on Symptoms of Depression and Social Anxiety in Adolescent Boys and Girls

**DOI:** 10.1007/s10964-023-01836-0

**Published:** 2023-08-17

**Authors:** Rebecca van Rijn, Nikki C. Lee, Miriam Hollarek, Hester Sijtsma, Reubs J. Walsh, Mariët van Buuren, Barbara R. Braams, Lydia Krabbendam

**Affiliations:** 1 Section of Clinical Developmental Psychology, Faculty of Behavioural and Movement Sciences, Vrije Universiteit Amsterdam, Amsterdam, The Netherlands; 2 Research Institute LEARN!, Amsterdam, The Netherlands; 3 Institute of Brain and Behavior Amsterdam, Amsterdam, The Netherlands; 4 Department of Developmental Psychology, Faculty of Social and Behavioural Sciences, Utrecht University, Utrecht, The Netherlands

**Keywords:** Pubertal timing, Early maturation, Social anxiety, Depression, Perceived popularity, Adolescence

## Abstract

Research has shown that adolescents – particularly girls – who mature relatively early often experience more internalizing problems. This effect is thought to be partially driven by psychosocial mechanisms, but previous research based relative pubertal maturation on complete samples or population standards, instead of considering the adolescents’ direct peer environment. In the current study the level of adolescents’ pubertal development was assessed relative to their classmates in order to examine relative pubertal maturation. The effects of adolescents’ relative pubertal status, and their perceived popularity, on symptoms of social anxiety and depression in adolescents were studied. All analyses were also performed for absolute pubertal maturation. Participants were 397 young adolescents (*M*_age_ = 13.06, *SD* = 0.36, 49.9% girls) at timepoint 1, and 307 (*M*_*age*_ = 14.08, *SD* = 0.36, 50.5% girls) at timepoint 2. A significant positive relationship was found between relative pubertal timing and symptoms of depression for girls but not boys. Social anxiety symptoms were not significantly related to relative pubertal timing in either sex. Relative pubertal maturation had no effect on change in or persistence of depressive and social anxiety symptoms one year later. The effects of the comparison with the immediate peer environment, did not seem to explain more variance in internalizing symptoms than the effects of early maturation.

## Introduction

Inter-individual variations in the timing of pubertal onset and development create a period during adolescence during which same-age and same-sex peers differ significantly with respect to several highly evident physical characteristics. These changes take place in a phase characterized by high vulnerability to peer evaluation (Beidel & Alfano, 2011; Carter et al., 2020). Relatively early maturation is often related with higher risk for internalizing symptoms (Copeland et al., 2010; Graber, 2013). In previous research relative pubertal timing has been determined based on population standards, absolute age cut-offs or using the means of an entire research sample (Blumenthal et al., 2011). These methods have provided relevant insights into the relationship between relatively early pubertal onset and internalizing problems, but do not consider the adolescents’ direct peer environment. As adolescents spend a lot of their time with their peers (Brown, 2004) and are sensitive to social comparison (Dijkstra et al., 2008; Parsons, 2008), it is also important to take the adolescents’ direct peer environment into consideration. Therefore, in the current study the level of adolescents’ pubertal development relative to their classmates was assessed. As adolescents in the same school class spend most of their time together, these school class mates are possibly a very salient comparison group (Reynolds & Juvonen, 2012). This method compares the pubertal status of same-sex classmates to determine whether an individuals’ pubertal development was relatively early, late or average. Then the effects of relative pubertal timing on internalizing problems (depressive symptoms and social anxiety symptoms), and the effect of perceived popularity on this relationship were tested.

### Relative Early Pubertal Maturation and Internalizing Psychopathology

Psychological explanations of the association between relatively early maturation and internalizing problems highlight two potential mechanisms, namely developmental readiness and maturational deviance (Ge & Natsuaki, 2009). The developmental readiness hypothesis suggests that early maturing individuals are at risk of internalizing problems, as they may not be cognitively ready for the emotional, social and physiological changes of puberty, such as emerging sexual activity and their environment treating them more like adults (e.g., Ge et al., 2003; Marceau et al., 2011). This may especially be the case for relatively early maturing girls, who may experience unwanted sexual attention, as well as negative responses from others regarding their physical appearance and emerging sexuality. These experiences may lead to the development of low self-esteem and symptoms of depression and anxiety (Deardorff et al., 2007; Detweiler et al., 2014). The maturational deviance hypothesis suggests that individuals that are either the first or the last of their peer group to undergo the pubertal transition must deal with the fact they look physically ‘deviant’ compared to their peers (Negriff & Susman, 2011). Relatively early maturing individuals are the first to experience body odor, pubic hair and acne vulgaris, which may lead to a fear of, and possibly exposure to, ridicule or social rejection (La Greca & Harrison, 2005). Particularly for girls, in whom signs of early pubertal development may be most obvious, it may be difficult to maintain friendships with same-sex peers with less advanced pubertal development (Petersen et al., 1991), which may lead to increased feelings of isolation (Craig et al., 2001), and low self-esteem, body satisfaction and body image (Negriff & Susman, 2011; Stice et al., 2001). In boys, it was found that early maturation can also be associated with depressive symptoms, though the findings are less consistent than for girls (Ge et al., 2001a; Mendle & Ferrero, 2012). Some research also points to potential negative effects of relatively late maturation in boys (Negriff & Susman, 2011; Graber et al., 2004).

### Effect of Peer Context in Relative Pubertal Timing

In sum, early pubertal maturation is associated with increased risk for depression and anxiety, with the strongest evidence for negative effects of early maturation in girls. However, less is known about the individual and contextual factors that may amplify or alleviate these effects (Skoog et al., 2016; Skoog & Stattin, 2014). Based on the psychosocial mechanisms of early pubertal maturation described by the developmental readiness and maturational deviance hypotheses, it can be surmised that one important factor is the peer context. Indeed, there is initial evidence that peer-related support or stress modifies the risk associated with early pubertal maturation (Winer et al., 2016; Benoit et al., 2013). A study found that early pubertal timing was related to depressive symptoms only in adolescents who experienced high levels of peer stress, not in adolescents who experienced low levels of peer stress (Conley & Rudolph, 2009). More concretely, a positive relationship between depressive symptoms and relatively early pubertal timing for individuals with a low reputation-based peer status (also referred to as perceived popularity) was found, but not for individuals with high peer status (Teunissen et al., 2011). This effect was present for both girls and boys. These findings suggest that high popularity may be a protective factor for the negative effects of relatively early maturation, as a source of status and esteem, whereas for already unpopular individuals, early pubertal timing can be a source of ridicule or ostracism (Teunissen et al., 2011). Research showed that early maturing girls are viewed as more popular among peers (Waylen & Wolke, 2004) and also themselves feel more popular among peers (McCabe & Ricciardelli, 2004). However, there are also studies that suggest that a high perceived popularity status may not help, but hurt. For girls, being popular and relatively early maturating may also cause unwanted sexual attention (Reynolds & Juvonen, 2011), more rumors being spread (Reynolds & Juvonen, 2011), and differential treatment (Mendle & Ferrero, 2012) or jealousy and resentment (Rose et al., 2011) by the adolescents’ environment. These suggest, on the other hand, that early maturing girls with higher perceived popularity would have a higher risk of depressive symptoms. Currently, no consensus has been reached in the literature.

Some studies have looked into the persistence of depressive symptoms associated with early pubertal maturation and showed that women that were early developers, had higher rates of psychosocial symptoms than women that were on-time developers (Graber et al., 2004). Ge et al. (2001b) report that early maturation is a more powerful predictor for depression and anxiety four years later than early life stressful events. Subclinical depressive symptoms can predict the presence of clinical episodes later in life, stating the importance to study subclinical symptoms as well (Byrne et al., 2019).

## The Present Study

Studies that investigate the effect of early pubertal timing in both boys and girls are still scarce, and studying relative pubertal maturation is generally based on the whole sample or population standards, instead of considering the adolescents’ direct peer environment. This study aims to address this gap by examining the effects of both relative pubertal timing based on the pubertal development of same-sex school classmates, and the effects of absolute pubertal maturation, on depressive and social anxiety symptoms using both concurrent and prospective analyses. Based on previous literature, the following hypotheses were formulated. Firstly, it was expected that relative early pubertal maturation (i.e., relatively more developed than classmates) is associated with more symptoms of depression and social anxiety, with stronger effects in girls than in boys. Second, it was hypothesized that high perceived popularity would be a protective factor in the relationship between relative pubertal maturation and symptoms of depression and social anxiety, with a stronger effect in girls than in boys. Third, it was expected that relative pubertal maturation predicts change in or persistence of symptoms of depression and social anxiety at the one-year follow-up measurement, with a stronger effect in girls than in boys. With this novel assessment of relative pubertal maturation, we aim to zoom in on the role of the peer context in the associations between early maturation and symptoms of depression and social anxiety. In order to relate the current findings to earlier research, all analyses were repeated using absolute pubertal maturation. No explicit hypothesis was formulated on how this novel assessment relates to the absolute pubertal maturation analyses.

## Methods

### Participants

The sample consisted of 397 adolescents (198 girls). Participants could self-identify as ‘boy’, ‘girl’ or ‘other’, but no participants in the current sample indicated themselves as ‘other’. Participants were in the first year of high school at time point 1 (T1) of the current study (*M*_*age*_ = 13.06 years, *SD* = 0.36), and in the second year of high school at time point 2 (T2; *M*_*age*_ = 14.08 years, *SD* = 0.36). The current sample was highly homogeneous, with 6 participants (1.5%) not born in the Netherlands. They were born in other countries in Europe, Central America, Asia and Middle-East.

This study was performed using a subsample of the #SOCONNeCT study, a longitudinal project investigating the development of social cognition during early adolescence (see also, e.g. Sijtsma et al., 2021; Walsh et al., 2023). During this project, participants were followed for the first three years of high school. Participants were recruited from seven mainstream high schools in the Netherlands, all pupils were enrolled in havo or VWO high school levels. These constitute the levels of education in the Netherlands with the highest achieving pupils academically, which make up around 50% of Dutch high school students (Central Bureau for Statistics, 2018). Data was collected within school classes, a group of 20 to 30 adolescents that belong to the same academic year and follow all courses together. They spend most of their school day together. Data was collected during school visits, with two waves of data collection roughly six months apart for the duration of the project, resulting in a total of six data collection waves. Only in second, fourth and sixth wave data regarding psychopathology were collected. In the current study, data from waves 2 and 4 (spring of the first and the second year of high school) were analyzed. In spring 2020 (start of COVID-19 pandemic) the sixth wave of data was collected online. Because of the unusual circumstances at that time, and potential effects on reported symptoms of social anxiety and depression, it was decided not to include wave 6 in this study.

**Fig. 1:**
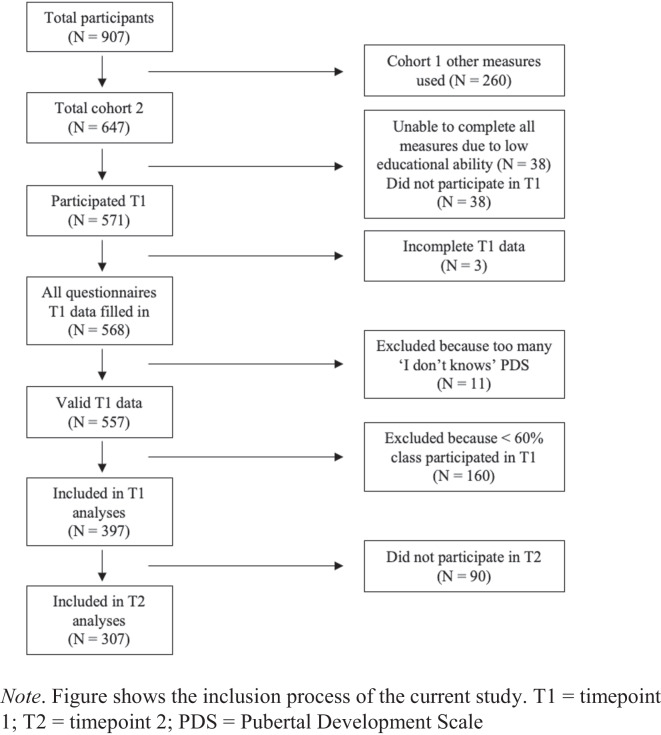
Flow chart of inclusion process.

In total, 907 adolescents (461 girls) were included in the #SOCONNeCT project. These participants were included in two different cohorts, and only the second cohort (N = 647) were administered all measures used in the current study, and therefore only these participants were included. Of these, the participants from one school (N = 38) were excluded, as they were unable to complete all measures due to low educational ability. A total of 38 participants did not participate in the second wave (T1 in the current study) of data collection (missed the second wave or joined the study later). In total, 571 participated in timepoint 1 (T1; the second wave of data collection in the #SOCONNeCT study). Three of those had incomplete data, meaning that they did not complete one of the following questionnaires, the Pubertal Development Scale, Social Anxiety Scale for Adolescents, Center for Epidemiological Studies Depression Scale for Children, and peer nominations. These participants were excluded. Another 11 participants were excluded based on their Pubertal Development Scale response (see below). Furthermore, participants were excluded from the current study if less than 60% of their same-sex classmates participated. To make sure popularity scores and relative pubertal maturation z-scores were reliable, a minimum of 60% percent of the pupils in a school class had to participate in the study in order for the school class to be included (Marks et al., 2013). This caused 160 participants to be excluded from the study. This resulted in the number of participants included in the first, cross-sectional analysis, N = 397. The participants belonged to 22 school classes, with participation rates per sex ranging from 60 to 100%. Of these participants, 90 did not participate during timepoint 2 ((T2; the fourth wave of data collection in the #SOCONNeCT study), leaving a total of 307 participants to be included in the second, prospective analysis. The participants that dropped out after T1 had higher depressive scores (*t*(395) = −0.270), *p* = 0.007), but did not differ on social anxiety scores (*t*(395) = 1.33, *p* = 0.186), absolute pubertal development scores (*t*(395) = 0.75, *p* = 0.455) and perceived popularity scores (*t*(395) = 1.67), *p* = 0.105). Figure 1 shows a flow chart of the inclusion process.

### Measures

#### Relative pubertal maturation

Relative pubertal maturation was based on scores of the Pubertal Development Scale (PDS; Petersen et al., 1988), a self-report questionnaire with items for both males and females, assessing growth spurts, skin changes and body hair changes, with specific items for females about breast development and onset of menarche, and specific items for males assessing facial hair growth and voice change. The questionnaire consists of five items for males and five items for females. Items are scored on a 4-point Likert scale. Answer options range from ‘no physical change’ (scored as 1) to ‘development seems complete’ (scored as 4), with an additional option ‘I don’t know’ (scored as 0). Whether or not onset of menarche has occurred is a yes/no question, with ‘no’ scored as 1 and ‘yes’ scored as 4. A minimum of four questions answered with a response other than ‘I don’t know’ was required to include participants in the analyses. The mean score was calculated using the sum score, divided by the number of answered questions (with other than ‘I don’t know’), which was either 4 or 5 (Braams et al., 2015). Final scores ranged from 1 to 4, with higher scores indicating a more progressed pubertal development. The Cronbach’s alpha values for the present sample were 0.67 for girls and 0.69 for boys. This is similar compared to other studies in which the Pubertal Development Scale is used, for example α = 0.61–0.75 (Díaz-Morales et al., 2014), α = 0.67–0.75 (Randler et al., 2009) and α = 0.72–0.74 (Greenlaef et al., 2014).

Relative pubertal maturation was calculated based on the average PDS score of the participating individuals per school class, and was calculated separately for boys and girls. Boys and/or girls in a school class were included in the analysis if more than 60% of their classmates of the respective sex participated. To clarify, for example for girls in a school class to be included, 60% of the girls in that school class should have been participating in the study. Z-scores were calculated using the average PDS score of the members of the same sex in the same school class, the standard deviation of the average PDS scores and the PDS score of the individual. Relative pubertal maturation was calculated using the PDS scores collected at T1. A higher z-score indicates a relatively more progressed pubertal development compared to their same-sex school classmates.

#### Depressive symptoms

Depressive symptoms were assessed using the Dutch translation of the Center for Epidemiological Studies Depression Scale for children (CES-DC; Weissman et al., 1980; Bouma et al., 2012), a 20-item questionnaire assessing depressed and positive affect, somatic and retarded activity and interpersonal aspects typically related to depressive symptomatology. CES-DC assesses behavior or feelings experienced during the past week. Examples of questions are: ‘I felt like crying’, ‘I felt like I couldn’t pay attention to what I was doing’ and ‘I didn’t sleep as well as I usually sleep’. Items are rated on a 4-point Likert scale, answer options ranged from ‘Not at all’ (0) to ‘A lot’ (3). Total scores range from 0 to 60, with higher scores resembling more feelings of depression. A score of 16 and higher is used as an indication of (sub)clinical levels of depression symptoms (Ensel, 1986; Bouma et al., 2012). Depression scores were collected at T1 and T2. The Cronbach’s alpha values for the present sample were 0.90 for T1 and 0.91 for T2.

#### Social anxiety symptoms

Social anxiety symptoms were assessed with the Dutch translation of the Social Anxiety Scale for Adolescents (SAS-A; La Greca & Lopez, 1998). The SAS-A consists of 22 items. Eighteen items assessing three domains of social anxiety and four filler items. Domains assessed are fear of negative evaluation (e.g. ‘I worry what other kids think of me’), social avoidance and distress in new situations (e.g. ‘I get nervous when I meet new kids’), and generalized avoidance and distress (e.g. I’m quiet when I’m with a group of people’). Items are rated on a 5-point Likert scale, ranging from ‘Not at all’ (1) to ‘All the time’ (5), according to extent to which each item describes them. Ratings for the 18 social anxiety items are summed to calculate a total score. Total scores range from 18 to 90. Scores of 50 or higher suggest clinically significant levels of social anxiety (La Greca, 1999). Social anxiety scores were collected at T1 and T2. The Cronbach’s alpha values for the present sample were 0.93 for both T1 and T2.

#### Perceived popularity

Peer nominations were used to assess perceived popularity (Cillessen & Mayeux, 2004). Participants were provided with a list of names of classmates participating in the #SOCONNeCT study. In response to two peer nomination questions, ‘Who in your school class is popular?’ and ‘Who in your school class is unpopular?’, participants were asked to nominate their classmates. Same-sex and other-sex nominations were permitted. The total number of nominations each participant received was summed, and standardized for school class size by dividing the summed number of nominations by school class size minus one. Perceived popularity was calculated by subtracting standardized ‘unpopular’ nominations from standardized ‘popular’ nominations (Cillessen & Mayeux, 2004). Values range from −1.0 to +1.0, with −1.0 resembling an unanimously unpopular status, and +1.0 an unanimously popular status. Perceived popularity scores were collected at T1.

### Procedure

Parents and participants received an information package at school and via email. After reading the information, participants and parents could return their consent form via mail or email. Parental consent and participant consent were obtained before study participation. Participants additionally filled in a paper consent form at the start of the study, during the first test wave. Participants could withdraw from participation at any moment, without giving a reason. The research protocol for the #SOCONNeCT study was approved by the VCWE (Vaste Commissie Wetenschap en Ethiek) of the Faculty of Movement and Behavioural Sciences of the Vrije Universiteit Amsterdam.

Data was collected during school visits. During the school visits participants individually filled in questionnaires on an iPad and completed several tasks on a laptop under supervision of trained researchers and students. Qualtrics XM software was used to administer the questionnaires. Schools received €7,50 per participant per wave which could be used for a joint school class activity.

#### Statistical Analysis

Scores for PDS (absolute pubertal maturation), SAS-A (symptoms of social anxiety), CES-DC (symptoms of depression) and perceived popularity scores were calculated as well as z-scores (relative pubertal maturation). Scores for perceived popularity and absolute pubertal maturation were mean centered before conducting analyses. Descriptive statistics were calculated to check assumptions of statistical tests and to check for outliers. One participant deviated more than three standard deviations from the mean of the depression score. Analyses were conducted both in- and excluding this participant, but as this did not change the pattern of results, only the results from the full sample analyses are reported.

All research questions were answered using multilevel models. Multilevel models are appropriate when data has a hierarchical structure, such as participants nested in school classes and schools, as is the case in the current sample. Intraclass correlation coefficients indicated that school could be disregarded as a level of the nested data, for both depression and social anxiety scores (ICC = 0; Koo & Li, 2016). School class and participant remained as levels. The analyses were performed in Rstudio, version 2023.03.0 (RStudio Team, 2020), using the lme4 and lmerTest packages (Bates et al., 2015; Kuznetsova et al., 2017).

For both outcome measures (social anxiety and depression), a model building procedure was used to test the hypotheses. Maximum likelihood estimation method was used to fit the models. At every step of the model building, fixed or random effects were added. With every step, the new model was compared with the previous model. Models were compared with the likelihood ratio test, and models were considered significantly better when p value was below 0.05. For completeness, Akaike information criterion (AIC) values and Bayesian information criterion (BIC) values were also shown. Lower values indicate better model fit. The best fitting model was selected and further estimated for boys and girls separately if the interaction between pubertal developmental and sex turned out significant. Highest finished parental level of education (ranging from 1 = finished primary school to 4 = finished tertiary education (higher professional education or university)) was added to separate model building procedures as a control variable. The models with parental level of education were compared to models without parental level of education with the likelihood ratio test, and if models were considered significantly better, the control variable was included in the final model. The model with parental level of education did not explain significantly more variance than the model without parental level of education for any of the model building procedures. Therefore, the control variable parental education was not included in the final models.

##### Pubertal maturation, depression and social anxiety symptoms and the moderating role of perceived popularity

The relationship between relative pubertal maturation (z-score), sex and symptoms of depression as well as the relationship between relative pubertal maturation (z-score), sex and symptoms of social anxiety were investigated using multilevel analyses. The relationship between absolute pubertal maturation (PDS score), sex and symptoms of depression and respectively social anxiety symptoms were also investigated with multilevel analyses. The model building procedure described below was performed for both depression and social anxiety symptoms as dependent variables, with both relative pubertal timing and absolute pubertal maturation as predictors. First, a null model with only a random intercept for group was fitted, without fixed effects. In Step 1, relative pubertal timing (in parallel models; absolute pubertal maturation) was added as a fixed effect. In Step 2, the two-way interaction between relative pubertal timing and sex was added as a fixed effect (and the lower order terms, i.e., the main effects of relative pubertal timing and sex). In Step 3, the three-way interaction between relative pubertal timing, sex and perceived popularity (and the lower order terms, i.e., the main effects of relative pubertal timing, sex and perceived popularity and the related two-way interactions) were added. In Step 4, the random slope of relative pubertal timing on the level of school class was added.

##### Pubertal maturation year 1 as a predictor of symptoms of depression and social anxiety year 2

The predictive value of relative pubertal maturation during the first year of high school for depressive and social anxiety symptoms during the second year of high school were investigated using multilevel analyses.

The model building procedure described below was performed for depressive symptoms at T2 as dependent variable, with both relative pubertal timing and absolute pubertal maturation as predictors. First, a null model with only a random intercept for group was fitted, without fixed effects. In Step 1, depressive symptoms during T1 was added as a fixed effect. In Step 2, the two-way interaction between depressive symptoms at T1 and relative pubertal maturation (in parallel models; absolute pubertal maturation) was added as a fixed effect (and the lower order terms, i.e., the main effects of depressive symptoms at T1 and relative pubertal timing). In Step 3, the three-way interaction between depressive symptoms, relative pubertal timing and sex (and the lower order terms, i.e., the main effects of depressive symptoms at T1, relative pubertal timing and sex and the related two-way interactions) were added. In Step 4, the random slope of depressive symptoms at T1 on the level of school class was added.

The model building procedure for social anxiety symptoms at T2 as dependent variable with both relative pubertal timing and absolute pubertal maturation as predictors is the same as described above for depressive symptoms.

## Results

### Descriptive Statistics

Descriptive statistics at T1 and T2 are reported for the total sample (N = 397 at T1; N = 307 at T2), and boys *(n* = 199 at T1; *n* = 152 at T2) and girls (*n* = 198 at T1; *n* = 155 at T2) separately in Table 1. In the current sample, t-tests indicated that symptoms of social anxiety and depression at both T1 and T2 were significantly higher in girls compared to boys (Table 1). Additionally, the absolute Pubertal Development Scale scores were also found to be significantly higher in girls compared to boys, indicating that girls in the current sample had progressed further in their pubertal transition than boys (Table 1). Other variables were not significantly different between boys and girls. Table 2 shows correlations between all model variables. Results for the whole sample show a strong correlation between social anxiety at T1 and perceived popularity, *r* = −0.209, *p* < 0.001, as well as between relative pubertal maturation and perceived popularity, *r* = 0.154, *p* = 0.002.

**Table 1:** Descriptive statistics

	Total sample (*N* = 397)			Boys (*n* = 199)			Girls (*n* = 198)			
	*M (SD)*	Min	Max	*M (SD)*	Min	Max	*M (SD)*	Min	Max	Cohen’s *d*
Age	13.06 (0.36)	11.67	14.21	13.06 (0.37)	11.67	14.21	13.07 (0.34)	12.07	14.06	−0.03
Social anxiety	38.41 (11.90)	18.00	89.00	36.42 (10.76)	18.00	76.00	40.40 (12.67)***	20.00	89.00	−0.34
Depression	14.30 (10.25)	0.00	55.00	11.73 (8.44)	0.00	55.00	16.88 (11.23)***	0.00	52.00	−0.52
Perceived popularity	−0.03 (0.49)	−1.00	1.00	0.01 (0.50)	−1.00	1.00	−0.07 (0.48)	−0.96	0.95	0.16
Relative pubertal maturation (Z-score)	0.005 (0.95)	−2.21	2.86	0.002 (0.96)	−2.21	2.86	0.008 (0.96)	−1.88	2.04	−0.01
Absolute pubertal maturation	2.26 (0.73)	1.00	4.00	1.93 (0.59)	1.00	4.00	2.60 (0.71)***	1.00	4.00	−1.01
Parental level of education	3.58 (0.65)	2.00	4.00	3.56 (0.65)	2.00	4.00	3.60 (0.63)	2.00	4.00	−0.06

	Total sample (N = 307)			Boys (*n* = 152)			Girls (*n* = 155)			
	*M (SD)*	Min	Max	*M (SD)*	Min	Max	*M (SD)*	Min	Max	Cohen’s *d*
Age	14.08 (0.36)	12.74	15.34	14.08 (0.34)	12.74	15.34	14.08 (0.34)	13.05	15.07	0.02
Social anxiety	39.98 (12.13)	18.00	85.00	37.29 (11.18)	18.00	82.00	42.63 (12.63)***	20.00	85.00	−0.45
Depression	13.36 (9.86)	0.00	45.00	10.93 (9.45)	0.00	45.00	15.75 (9.70)***	1.00	41.00	−0.50

Table describes total sample, and girls and boys separately. Measurements collected during the first year of high school are noted as T1, measurements collected during the second year of high school noted as T2. T-tests indicate a significant difference in social anxiety and depressive symptoms between boys and girls, both in T1 and T2, and between absolute pubertal maturation between boys and girls

*M* mean; *SD* standard deviation

***p < 0.001

**Table 2:** Correlations for all model variables

Total sample (*N* = 397 at T1; *N* = 307 at T2)						
	1.	2.	3.	4.	5.	6.
1. Social anxiety symptoms T1						
2. Depressive symptoms T1	0.572***					
3. Perceived popularity	−0.209***	−0.059				
4. Relative pubertal maturation	0.041	0.127*	0.154**			
5. Absolute pubertal maturation	0.101*	0.236***	0.081	0.839***		
6. Social anxiety symptoms T2	0.635***	0.498***	−0.278***	0.038	0.147*	
7. Depressive symptoms T2	0.308***	0.546***	−0.071	0.107	0.258***	0.584***

Boys (*n* = 199 at T1; *n* = 152 at T2)						
	1.	2.	3.	4.	5.	6.
1. Social anxiety symptoms T1						
2. Depressive symptoms T1	0.550***					
3. Perceived popularity	−0.275***	−0.130				
4. Relative pubertal maturation	−0.012	0.004	0.097			
5. Absolute pubertal maturation	−0.042	0.015	0.096	0.958***		
6. Social anxiety symptoms T2	0.598***	0.515***	−0.328***	0.96	0.123	
7. Depressive symptoms T2	0.329***	0.616***	−0.051	0.123	0.165*	0.602***

Girls (*n* = 198 at T1; *n* = 155 at T2)						
	1.	2.	3.	4.	5.	6.
1. Social anxiety symptoms T1						
2. Depressive symptoms T1	0.578***					
3. Perceived popularity	−0.158*	0.010				
4. Relative pubertal maturation	0.088	0.264***	0.219**			
5. Absolute pubertal maturation	0.117*	0.230***	0.193**	0.950***		
6. Social anxiety symptoms T2	0.644***	0.431***	−0.219**	0.000	0.000	
7. Depressive symptoms T2	0.253***	0.461***	−0.083	0.080	0.089	0.494***

Table describes correlations for all model variables, for total sample and girls and boys separately

*p ≤ 0.05; **p ≤ 0.01; ***p ≤ 0.001

### Effects of Relative and Absolute Pubertal Maturation, Sex and Perceived Popularity on Depressive Symptoms

#### Relative pubertal maturation

The model building procedure showed that the Step 2 model was the final model. Table 3 presents the results of the model building procedure. The final model includes the two-way interaction between relative pubertal maturation and sex and the main effects of relative pubertal timing and sex as a fixed effect, and the random intercept for group (full description of final model in Table 4). The results of the likelihood ratio test when comparing this model to the previous (Step 1) was χ^2^(2) = 39.25; *p* < 0.001. Step 3, the model including the three-way interaction between relative pubertal timing, sex and perceived popularity, was approaching significance (χ^2^(4) = 8.00; *p* = 0.092), but was not significantly better than the Step 2 model.

**Table 3:** The AIC and BIC values of the model building procedures for investigating the effect of relative pubertal timing on depressive symptoms and social anxiety symptoms. Absolute pubertal maturation between brackets

Internalizing problem	Null model		Model in Step 1		Model in step 2		Model in step 3		Model in step 4	
	AIC	BIC	AIC	BIC	AIC	BIC	AIC	BIC	AIC	BIC
Depression	2979.5 (2979.5)	2991.5 (2991.5)	2971.0 (2949.8)	2986.9 (2965.8)	**2935.7** **(2933.2)**	**2959.6** **(2957.2)**	2935.7 (2932.9)	2975.6 (2972.8)	2939.7 (2936.4)	2987.5 (2984.2)
Social anxiety	**3098.1** (3098.1)	**3110.0** (3110.0)	3098.6 (3093.5)	3114.6 (3109.4)	3090.3 (3088.5)	3114.2 (3112.4)	3069.0 **(3066.8)**	3108.8 **(3106.7)**	3072.9 (3070.6)	3120.7 (3118.4)

Table describes the Akaike information criterion and Bayesian information criterion values of the model building procedures for the effect of relative pubertal timing on depressive symptoms and social anxiety symptoms. Null model contains the random intercept for school class, without fixed effects. In step 1, relative pubertal maturation is added as a fixed effect (absolute pubertal maturation in parallel models). In step 2, the interaction relative pubertal maturation and sex was added as fixed effect (with lower order terms). In step 3, the interaction between relative pubertal maturation, sex and perceived popularity and all lower order terms were added. In step 4, the random slope of relative pubertal maturation on level of school class was added. The AIC and BIC values for the model building process for the effect of absolute pubertal timing in brackets. Final models are printed in bold

**Table 4:** Final model for predicting depressive symptoms with relative pubertal maturation (absolute pubertal timing between brackets)

Total sample (*N* = 397)						
	Beta coefficient	Std. dev./ SE	*t*-value	*p*-value	95% CI	
					Lower	Upper
Random effects						
Intercept group		0.00 (0.00)			0.00 (0.00)	2.17 (2.17)
Residual		9.66 (9.64)			8.98 (8.96)	10.32 (10.29)
Fixed effects						
Intercept	11.72 (11.66)	0.68 (0.78)	17.21 (14.91)	<0.001*** (<0.001***)	10.39 (10.13)	13.07 (13.19)
Relative pubertal timing	−0.07 (−0.20)	0.72 (1.16)	−0.10 (−0.17)	0.919 (0.863)	−1.48 (−2.47)	1.33 (2.96)
Sex	5.13 (3.57)	0.97 (1.09)	5.29 (3.29)	<0.001*** (0.001***)	3.23 (1.45)	7.02 (5.69)
Relative pubertal timing * sex	3.63 (5.18)	1.02 (1.51)	3.56 (3.44)	<0.001*** (<0.001***)	1.64 (2.24)	5.62 (8.12)

*Std. dev.* standard deviation, *SE* standard error, *CI* confidence interval

***p ≤ 0.001

**Fig. 2:**
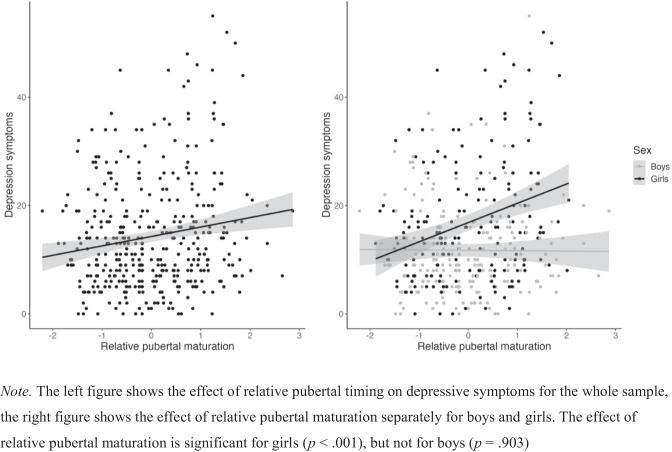
The effect of relative pubertal timing on depressive symptoms for the whole sample and stratified for sex.

The final model indicates a significant interaction between relative pubertal maturation and sex, so the effects of relative pubertal maturation were studied for boys and girls separately. Model descriptions can be found in Table 5. Results indicate that relative pubertal timing is a significant predictor for depressive symptoms in girls (*p* < 0.001), but not in boys (*p* = 0.903). This is visualized in Fig. 2.

**Table 5:** Separate analyses by sex for investigating the influence of relative pubertal maturation on depressive symptoms (absolute pubertal timing between brackets)

Boys (*n* = 199)						
	Beta coefficient	Std. dev./ SE	*t*-value	*p*-value	95% CI	
					Lower	Upper
Random effects						
Intercept group		2.81 (2.81)			0.80 (0.81)	4.67 (4.68)
Residual		8.04 (8.04)			7.25 (7.25)	8.93 (8.93)
Fixed effects						
Intercept	11.92 (11.82)	0.87 (0.93)	13.77 (12.77)	<0.001*** (<0.001***)	10.20 (10.00)	13.69 (13.69)
Relative pubertal timing	−0.07 (−0.27)	0.60 (0.98)	−0.12 (−0.28)	0.903 (0.783)	−1.25 (−2.20)	1.10 (1.67)

Girls (*n* = 198)						
	Beta coefficient	Std. dev./ SE	t-value	*p*-value	95% CI	
					Lower	Upper
Random effects						
Intercept group		0.00 (0.00)			0.00 (0.00)	2.71 (2.48)
Residual		1.74 (10.68)			9.71 (9.66)	11.83 (11.77)
Fixed effects						
Intercept	16.86 (11.82)	0.76 (0.84)	22.09 (18.20)	<0.001*** (<0.001***)	15.36 (13.60)	18.35 (16.88)
Relative pubertal timing	3.55 (4.98)	0.80 (1.07)	4.44 (4.68)	<0.001*** (<0.001***)	1.98 (2.89)	5.12 (7.07)

*Std. dev.* standard deviation, *SE* standard error, *CI* confidence interval

***p ≤ 0.001

#### Absolute pubertal maturation

The same model building procedure was performed with absolute pubertal maturation (instead of relative pubertal maturation) and results were very similar. Table 3 shows the results of the model building procedure, between brackets. The model building procedure indicates that the Step 2 model was the final model, as for relative pubertal timing. This model contains the two-way interaction between absolute pubertal timing and sex, the main effects of pubertal timing and sex, and the random intercept for group. The full description of the model is shown in Table 4, between brackets. The results of the likelihood ratio test when comparing this model to the previous (Step 1) was χ^2^(2) = 20.58; *p* < 0.001. Step 3, the model with the three-way interaction between absolute pubertal timing, sex and perceived popularity, was approaching significance (χ^2^(4) = 8.32; *p* = 0.080), but was not significantly better than the Step 2 model.

The final model indicates a significant interaction between absolute pubertal maturation and sex, the effects of absolute pubertal maturation were studied for boys and girls separately. Model descriptions can be found in Table 5, between brackets. Results indicate that absolute pubertal timing is a significant predictor for depressive symptoms in girls (*p* < 0.001), but not in boys (*p* = 0.783).

### Effects of Relative and Absolute Pubertal Maturation, Sex and Perceived Popularity on Social Anxiety Symptoms

#### Relative pubertal maturation

The model building procedure showed that adding relative pubertal maturation as a fixed effect did not explain significantly more variance than the null model, containing only the random intercept for groups. The results of the likelihood test between the null model and the Step 1 model was χ^2^(1) = 1.45; *p* = 0.229. Table 3 shows the results of the model building procedure.

#### Absolute pubertal maturation

**Fig. 3:**
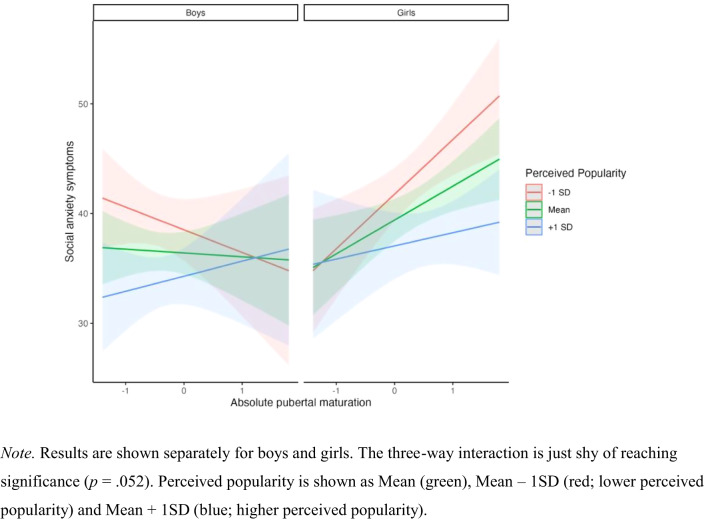
Visualization of the trend-level three-way interaction between absolute pubertal maturation, sex and perceived popularity predicting social anxiety symptoms.

The same model building procedure was performed with absolute pubertal maturation (instead of relative pubertal maturation). Table 3 shows the results of the model building procedure, between brackets. The model building procedure indicates that the Step 3 model was the final model. The results of the likelihood ratio test when comparing this model to the previous (Step 2) was χ^2^(4) = 29.68; *p* < 0.001. The final model contains the three-way interaction between absolute pubertal timing, sex and perceived popularity, the lower order terms, i.e., the main effects of absolute pubertal timing, sex and perceived popularity and the related two-way interactions. The full description of the model can be found in Table 6. The main effects of sex (*p* = 0.023) and perceived popularity (*p* = 0.027) were found to be significant predictors for social anxiety symptoms. The three-way interaction between absolute pubertal timing, sex and perceived popularity was not significant (*p* = 0.052), but visualized in Fig. 3 to depict the trend. The interaction between absolute pubertal timing and sex was also not significant (*p* = 0.055), therefore the separate analyses for boys and girls were not conducted.

**Table 6:** Final model for predicting social anxiety symptoms with absolute pubertal maturation

Total sample (*N* = 397)						
	Beta coefficient	Std. dev./ SE	*t*-value	*p*-value	95% CI	
					Lower	Upper
Random effects						
Intercept group		1.49			0.00	3.12
Residual		11.26			10.41	12.00
Fixed effects						
Intercept	36.41	0.98	37.04	<0.001***	34.49	38.31
Absolute pubertal timing	−0.35	1.37	−0.26	0.798	−3.01	2.31
Sex	3.00	1.32	2.28	0.023*	0.44	5.56
Perceived popularity	−4.31	1.94	−2.22	0.027*	−8.08	−0.48
Absolute pubertal timing * sex	3.44	1.79	1.92	0.055	−0.04	6.91
Absolute pubertal timing * perceived popularity	3.51	2.98	1.18	0.239	−2.30	9.29
Sex * perceived popularity	−0.50	2.81	−0.18	0.859	−6.03	4.95
Absolute pubertal timing * sex * perceived popularity	−7.36	3.78	−1.95	0.052	−14.71	0.01

*Std. dev.* standard deviation, *SE* standard error, *CI* confidence interval

*p ≤ 0.05; ***p ≤ 0.001

### Prospective Effects of Pubertal Maturation on Internalizing Symptoms

Finally, it was investigated whether relative pubertal maturation in the first year of high school (T1) explained persistence or change in depression and social anxiety scores in the second year of high school (T2) beyond T1 depression and social anxiety scores. For the predictive value of relative pubertal maturation, the year one values for symptoms of depression and social anxiety were added to the respective models to control for T1 scores in these analyses.

For depressive symptoms, the model building procedure showed that the Step 1 model was the final model, in both relative pubertal maturation and absolute pubertal timing. Table 7 presents the results of the model building procedure. The final model includes only the main effect of depressive symptoms in T1. Because neither relative pubertal maturation nor absolute pubertal maturation are in the model, this final model is used to present the results for depressive symptoms without differentiating between relative and absolute pubertal maturation. Table 8 describes the full model. The results of the likelihood ratio test when comparing this model to the previous (null model) was χ^2^(1) = 80.77; *p* < 0.001. It can be concluded that adding either relative pubertal maturation or absolute pubertal timing to the model could not explain more variance than the model with a fixed effect of depressive symptoms at T1 and the random intercept for groups.

**Table 7:** The AIC and BIC values of the model building procedures for investigating whether relative pubertal timing in the first year of high school (T1) explained persistence or change in depressive symptoms and social anxiety symptoms in the second year of high school (T2)

Internalizing problem	Null model		Model in Step 1		Model in step 2		Model in step 3		Model in step 4	
	AIC	BIC	AIC	BIC	AIC	BIC	AIC	BIC	AIC	BIC
Depression	2271.3 (2271.3)	2282.5 (2282.5)	**2192.6** **(2192.6)**	**2207.5** **(2207.5)**	2195.7 (2192.9)	2218.0 (2215.2)	2192.3 (2190.2)	2229.0 (2227.5)	2195.4 (2193.1)	2240.1 (2237.8)
Social anxiety	2406.0 (2406.0)	2417.2 (2417.2)	**2254.3** **(2254.3)**	**2269.2** **(2269.2)**	2258.0 (2255.0)	2280.4 (2277.4)	2256.8 (2255.9)	2294.1 (2293.2)	2252.8 (2259.7)	2297.5 (2304.4)

Table describes the Akaike information criterion and Bayesian information criterion values of the model building procedures for the persistence of relative pubertal timing on depressive symptoms and social anxiety symptoms over a year. Null model contains random intercept for school class. In step 1, depressive symptoms (respectively social anxiety symptoms) at T1 was added as fixed effect. In step 2, interaction between depressive symptoms and relative pubertal timing (absolute pubertal maturation in parallel models) was added as a fixed effect (with lower order terms). In step 3, interaction between depressive symptoms, relative pubertal maturation and sex and all lower order terms were added. In step 4, the random slope of depressive symptoms at T1 on the level of school class was added. The AIC and BIC values for the model building process for the effect of absolute pubertal timing in brackets. Final models are printed in bold

Absolute pubertal maturation between brackets

**Table 8:** Final models for investigating whether relative pubertal timing in the first year of high school (T1) explained persistence or change in depressive symptoms and social anxiety symptoms in the second year of high school (T2)

Depressive symptoms T2						
	Beta coefficient	Std. dev./ SE	*t*-value	*p*-value	95% CI	
					Lower	Upper
Random effects						
Intercept group		2.22			0.72	3.69
Residual		8.32			7.67	9.03
Fixed effects						
Intercept	6.80	0.99	6.90	<0.001***	4.87	8.76
Depressive symptoms T1	0.49	0.05	9.61	<0.001***	0.39	0.59

Social anxiety symptoms T2						
	Beta coefficient	Std. dev./ SE	*t*-value	*p*-value	95% CI	
					Lower	Upper
Random effects						
Intercept group		2.00			0.00	3.59
Residual		9.26			8.53	10.05
Fixed effects						
Intercept	15.51	1.87	8.27	<0.001***	11.83	19.17
Social anxiety symptoms T1	0.63	0.05	14.09	<0.001***	0.54	0.72

*Std. dev.* standard deviation, *SE* standard error, *CI* confidence interval

***p ≤ 0.001

For social anxiety symptoms, the model building procedure also showed that the Step 1 model was the final model. This was the case for both relative pubertal maturation and absolute pubertal timing. Table 7 presents the results of the model building procedure. The final model includes only the main effect of social anxiety symptoms in T1. Because neither relative pubertal maturation nor absolute pubertal maturation are in the model, this final model is used to present the results for social anxiety symptoms without differentiation. Table 8 describes the full model. The results of the likelihood ratio test when comparing this model to the previous (null model) was χ^2^(1) = 153.77; *p* < 0.001. It can be concluded that adding either relative pubertal maturation or absolute pubertal timing to the model could not explain more variance than the model with a fixed effect of social anxiety symptoms at T1 and the random intercept for groups.

## Discussion

Previous research focusing on relative pubertal timing did not consider adolescents’ direct peer environment in their methods. In this study, a novel approach was used to investigate the effects of early pubertal maturation relative to the direct peer group on internalizing problems. All analyses were also performed for absolute pubertal maturation. For symptoms of depression, the results of the concurrent analysis showed early pubertal maturation predicted depression scores in girls, but not in boys. Perceived popularity could not significantly explain additional variance. Absolute pubertal maturation revealed the same results, suggesting that more progressed absolute pubertal maturation predicted more depressive symptoms in girls, not boys. Relative early pubertal maturation had no effect on change in or persistence of depression scores at one-year follow-up, and neither did early absolute pubertal maturation. In contrast to the expectations, no effect of relative early pubertal maturation on symptoms of social anxiety was found. For absolute pubertal maturation, the model with the three-way interaction between absolute pubertal maturation, sex and perceived popularity (and the underlying two-way interaction and separate main effects) and the random intercept for group could best explain social anxiety symptoms. The three-way interaction itself just failed to reach significance. The main effects of perceived popularity and sex did significantly explain social anxiety symptoms. Relative early pubertal maturation and absolute pubertal maturation had no effect on change in or persistence of social anxiety scores at one-year follow-up. The novel assessment of relative pubertal maturation as investigated in this study, did not seem to explain more variance in social anxiety symptoms than the effects of early maturation.

### Effects of Relative and Absolute Pubertal Maturation, Sex and Perceived Popularity on Depressive Symptoms

Partly in line with the expectations and previous literature, early relative pubertal maturation was associated with higher depression scores in girls but not in boys (Graber et al., 2004; Joinson et al., 2012). As suggested by the maturational deviance hypothesis, the visible, physical changes may be linked to difficulties maintaining friendships with same-sex peers, disapproval among peers as a result of social comparison and decreasing self-esteem and body image in girls (Craig et al., 2001; Teunissen et al., 2011). These effects may contribute to the higher prevalence of depressive symptoms in relatively early maturing girls. The current findings also match the developmental readiness hypothesis, which suggests that early maturing individuals may not be ready for the emotional, social and physiological changes of puberty (e.g., Ge et al., 2003; Marceau et al., 2011; Petersen & Taylor, 1980). This might be especially relevant for relatively early maturing girls, who may experience unwanted sexual attention, as well as negative responses from others regarding their physical appearance and emerging sexuality. These experiences may contribute to the higher prevalence of depressive symptoms in relative early maturing girls (Deardorff et al., 2007; Detweiler et al., 2014).

The model building procedure indicated that adding perceived popularity as a fixed effect, in a model with the three-way interaction between relative pubertal maturation, sex and perceived popularity (the model also contained the lower-order two-way interactions and the separate main effects) just failed to explain significantly more variance than the model without perceived popularity. Therefore, it can be concluded that perceived popularity does not significantly influence the relationship between relative pubertal maturation and depressive symptoms, contrary to the hypothesis. Some earlier research indicated that perceived popularity could play a protective role in the relationship between relative pubertal maturation and depressive symptoms (Teunissen et al., 2011; Conley & Rudolph, 2009), but with research proposing the opposite effect (Reynolds & Jovonen, 2011; Rose et al., 2011), no consensus is reached in literature. Unfortunately, results from the current study cannot offer evidence to clarify this question.

The results for absolute pubertal maturation were very similar to those of relative pubertal timing. The model with the two-way interaction between relative pubertal timing and sex (and separate main effects) best explained depressive symptoms. And similar to relative pubertal maturation, for girls, absolute pubertal maturation seemed to predict depressive symptoms, while for boys this effect was not found. Similar to relative pubertal timing, the model with perceived popularity (the three-way interaction, lower-order two-way interactions and the main effects) could not explain more variance than the final model with two-way interaction by a small margin. For depressive symptoms, there seemed to be no difference between calculating the relative pubertal maturation based on pubertal development scores of same-sex school classmates and using pubertal development scores directly in the analyses. The suggested effects of pubertal development on depressive symptoms might be strongly influenced by biological and hormonal changes (Ge & Natsuaki, 2009; Detweiler et al., 2014), which could why in the current results there seems to be no apparent difference between investigating relative and absolute pubertal maturation.

### Effects of Relative and Absolute Pubertal Maturation, Sex and Perceived Popularity on Social Anxiety Symptoms

Similar to symptoms of depression, we hypothesized a significant relationship between relative pubertal maturation and social anxiety symptoms because embarrassing social interactions, increased self-consciousness and the fear of social rejection and disapproval from peers could cause relatively early maturing individuals to be more anxious. Contrary to the expectations, relative pubertal maturation did not seem to significantly predict social anxiety symptoms.

When analyses were performed with absolute pubertal maturation, the model building process revealed that the model including the three-way interaction between absolute pubertal timing, sex and perceived popularity, the lower order terms, i.e., the main effects of absolute pubertal timing, sex and perceived popularity and the related two-way interactions was the best fit to explain social anxiety symptoms. The main effects of sex and perceived popularity did significantly predict social anxiety symptoms. In line with the literature, girls reported more social anxiety symptoms than boys, and high perceived popularity was associated with fewer social anxiety symptoms (Henricks et al., 2021). The three-way interaction itself was not significant, by a small margin. Therefore, the effects of perceived popularity should be interpreted with caution. Visualization showed high popularity indeed appeared to buffer against the risk for social anxiety symptoms in early maturing girls. This finding is in line with literature on the effects of pubertal timing and perceived popularity on depressive symptoms. For example, it has been reported that low peer stress protected against symptoms of depression in early maturing girls and in late maturing boys (Conley & Rudolph, 2009). Examples of peer stress are peer victimization, social exclusion and low support or quality of friendships, which might be higher in adolescents with lower perceived popularity and could be related to social anxiety symptoms as well. Given that our study was unable to show significant three-way interactions with perceived popularity future research is encouraged to investigate whether a larger sample size could confirm the tentative buffering effect of high popularity on the risk for social anxiety in early maturing girls.

Whereas for depressive symptoms the models with relative pubertal maturation and absolute pubertal maturation were similar, for social anxiety the results were quite different. The novel assessment of relative pubertal maturation as investigated in this study, did not seem to explain more variance in social anxiety symptoms than the effects of early maturation. This could mean that the effects of early pubertal timing go beyond the comparing adolescents do within their school class. It has been suggested that peers from outside their school environment could also have strong effects on individuals’ development (Stattin et al., 2011). Therefore research focusing on classroom peers could miss potentially important peers (Stattin et al., 2011). This, together with the growing use of social media and as a result thereof increased opportunity for comparisons with peers and celebrities on the internet, should be considered in future research investigating the effects of relative pubertal maturation.

### Prospective Effects of Pubertal Maturation on Internalizing Symptoms

Finally, early pubertal maturation had no effect on change in or persistence of symptoms of depression or social anxiety one year later. Absolute pubertal maturation also had no effect on change in or persistence of symptoms of depression or social anxiety one year later. This could possibly be explained by the fact that the average age of girls participating in this study was 13, whereas in biologically early maturating girls, menarche can occur as early as age 10 or 11 (Papadimitriou et al., 2008). The negative effects of relative early maturation could be stronger at younger ages, and the risk of these negative effects could be more prominent at younger ages than in the older age group investigated in the current study. Literature even suggests that the link between pubertal timing and internalizing problems is temporary, and disappears over the course of adolescent development (Joinson et al., 2013). Boys typically start developing one year later, which was confirmed by the lower absolute pubertal maturation scores compared to girls. The boys in the current sample therefore have a more suitable age range to study the effect of relatively early pubertal maturation. For boys, the results from the concurrent analyses indicated there is no negative effect of early pubertal maturation on internalizing symptoms during this period of adolescence. There is literature indicating that relatively late pubertal maturation can cause problems in boys (Negriff & Susman, 2011; Graber et al., 2004), but research is not consistent (Ge et al., 2001a; Mendle & Ferrero, 2012). In sum, there seems to be no significant persistent effect of relative and absolute pubertal maturation on internalizing symptoms. This may be caused by the fact that the link between relatively early pubertal maturation and internalizing problems might not exist for boys, and the effect might not be persistent over time in girls. In line with the literature, the results of the analysis did show high persistence of both depression and social anxiety symptoms, with baseline scores showing substantial associations with scores at follow up (Blanco et al., 2011; Patten et al., 2001).

### Limitations and Future Directions

This study should be interpreted in light of several limitations. First, assessment of pubertal status was based on self-report rather than physician-ratings or solely reported age of menarche. However, using the PDS (Petersen et al., 1988) has several benefits: it has a male and female equivalent questionnaire version, assesses physical changes both caused by adrenarche and gonadarche pubertal developmental periods, is non-invasive and easily administered in school settings. Previous research has shown PDS has high internal consistency (Negriff & Susman, 2011; Koopman-Verhoeff et al., 2020). In the current study, PDS has an internal consistency of 0.67 and 0.69 (Cronbach’s alpha), which is considered sufficient, but not high. Many other studies have published results with similar internal consistencies (Díaz-Morales et al. 2014; Randler et al., 2009; Greenlaef et al., 2014). A highly consistent, comparable measure to test pubertal development for both boys and girls without the need for a physician would be of great value to the field.

The analyses of this study resulted in multiple analyses in p-values of <0.1, but not of <0.05, which could indicate insufficient power. However, post-hoc sensitivity analyses indicated that with the current sample size, effect sizes similar to those found in literature, although often small, could be detected (Ullsperger & Nikolas, 2017; Smith-Woolley et al., 2017). Furthermore, in the current study, participants that dropped out between T1 and T2, were found to have higher depressive symptoms compared to the individuals that participated in both T1 and T2. Although it cannot be stated with absolute certainty, it is possible that as a result of participants with higher depressive scores dropping out, the current effects of relative pubertal maturation on depressive symptoms might be dampened. For example, removing those most susceptible to depressive symptoms may remove those for whom depressive symptoms are most likely to increase due to early maturation. No participants actively withdrew from the study, and over the course of the study there was no indication that participants were unavailable during data collection for any reasons other than absence from school on the day of testing. However, it is possible that these absences may be due to illness, and potentially participants with more depressive symptoms may have missed school more often. Therefore, for future research, it would be beneficial to try and improve study adherence to minimize study dropout and when possible to check for reason of absence.

Additionally, attention should also be paid to the diversity of the research sample. The current sample consisted mainly of Caucasian adolescents and future research could benefit from a more diverse sample, as there are cultural differences regarding idealized female body type (Mendle et al., 2007; Negriff & Susman, 2011). The ideas about idealized female body type can influence the self-esteem of the newly developing girls and, concurrently, their risk of internalizing problems (Mendle et al., 2007; Negriff & Susman, 2011). Previous research has indicated that especially the Western cultural concept of an ideal body, focused on a more slender or thin body, can make girls more susceptible to symptoms of depression as a result of early pubertal development (Negriff & Susman, 2011). Moreover, in the current study, there was little heterogeneity in parental level of education and it was generally high. In light of the studied this topic, this should be considered a limitation, since literature indicates that factors such as social economic status can influence pubertal maturation and therefore have an effect on internalizing symptoms (Mendle et al., 2007; Nadeem & Graham, 2005). There are several different ways to operationalize social economic status and in this study, parental level of education is used as proxy for social economic status (American Psychological Association, 2007). Investigating a more diverse sample could gain insights on the relationship between relatively early pubertal maturation and internalizing problems.

A possibility to further investigate the effects of relative pubertal timing, rather than the z-score method based on same-sex school class-mates, is to use the assessment with self-perceived pubertal maturation. The adolescent is then asked whether they perceive themselves as early, on-time or late maturers (Mendle & Ferrero, 2012; Graber et al., 2004). Although these subjective estimations of their relative maturation may not be completely biologically accurate, self-perceptions may well tap into the psychosocial effects of relatively early and late maturation (Graber et al., 2004), even more so than the self-report biological-oriented questions of the PDS. The use of self-perception assessment of pubertal timing was also offered by Reynolds and Juvonen (2012), claiming that it could be particularly useful to assess the feeling of being ‘different’ from peers in terms of pubertal development, which may contribute to the development of psychological symptoms. With this approach, one could also try to disentangle the possibility that suffering from internalizing problems may affect adolescents’ perceptions of their pubertal timing. Within the current study, it was not possible to investigate this.

### Implications

The current findings have the potential to inform policy and practice. Depressive disorder is a common mental health problem in adolescents, with a prevalence of around 4–5% in mid adolescence (Costello et al., 2005), with strong female preponderance of 2:1 (Hyde et al., 2008). Research shows that subclinical depressive symptoms can predict the presence of clinical episodes later in life, stating the importance of studying subclinical symptoms as well (Byrne et al., 2017). Similar to depression, prevalence of anxiety disorders increases during early adolescence compared to childhood (Beidel et al., 2007). It was shown that 54% of all girls experience high levels of depressive and anxiety symptoms during the second half of adolescence (Patton et al., 2014). The current findings suggest that early maturing girls have a higher risk of developing depressive symptoms, which makes this group important targets for preventative interventions. Additionally, the findings also indicate that girls and individuals with lower perceived popularity have a higher risk of developing social anxiety symptoms. For these individuals, interventions based on emotional and social skill training could be useful (Collaborative for Academic, Social and Emotional Learning, 2014; Skoog et al., 2016). Giving schools and teachers more knowledge about these risk factors, could also help signaling problems in time.

## Conclusion

Relatively early maturating individuals often experience more internalizing problems. Because the psychological effects of early maturation are thought to be driven by social comparison, a measure of pubertal maturation relative to the immediate peer group was used. The school class-centered data collection created the possibility to use this novel approach. In the current study, the level of adolescents’ pubertal development relative to their classmates was assessed as a novel approach to relative pubertal maturation. All analyses were also performed for absolute pubertal maturation. Concurrent analyses showed that for adolescent girls, but not for boys, a significant positive relationship was found between early pubertal maturation and depressive symptoms. Furthermore, results indicated no significant relationship between symptoms of social anxiety and relative pubertal maturation in either boys or girls. There was tentative evidence that high perceived popularity could be a protective factor for social anxiety symptoms in early absolute pubertal maturation in girls, although results should be interpreted with care. Early maturation did not contribute to change in or persistence of depression and social anxiety symptoms. With more knowledge about the contextual risk factors for developing depressive or social anxiety symptoms, targeted preventative interventions can be used. The effects of the comparison with the immediate peer environment, did not seem to explain more variance in internalizing symptoms than the effects of early maturation.
